# Nicotinamide phosphoribosyltransferase as a biomarker for the diagnosis of infectious pleural effusions

**DOI:** 10.1038/s41598-021-00653-4

**Published:** 2021-10-26

**Authors:** Jing Huang, Lun Guo, Hong-Wei Kang, Dan Lv, Wei Lin, Chao-Fen Li, Xue-Qin Huang, Qun-Li Ding

**Affiliations:** 1 Department of Pharmacy, The Affiliated Hospital of Medical School of Ningbo University, Ningbo, 315020 China; 2 Department of Pulmonary and Critical Care Medicine, The Affiliated Hospital of Medical School of Ningbo University, Ningbo, 315020 China; 3 Department of Chemical Biology and Clinical Laboratory, The Affiliated Hospital of Medical School of Ningbo University, Ningbo, 315020 China; 4 School of Business, Zhejiang Pharmaceutical College, Ningbo, 315100 China; 5 Department of Chemical Biology and Clinical Laboratory, Ningbo Ninth Hospital, Ningbo, 305020 China; 6 Zhejiang Key Laboratory of Pathophysiology, Medical School of Ningbo University, Ningbo, 315211 China

**Keywords:** Biomarkers, Infectious diseases, Respiratory tract diseases

## Abstract

Nicotinamide phosphoribosyltransferase (NAMPT) has been reported to be involved in infectious diseases, but it is unknown whether it plays a role in infectious pleural effusions (IPEs). We observed the levels of NAMPT in pleural effusions of different etiologies and investigated the clinical value of NAMPT in the differential diagnosis of infectious pleural effusions. A total of 111 patients with pleural effusion were enrolled in the study, including 25 parapneumonic effusions (PPEs) (17 uncomplicated PPEs, 3 complicated PPEs, and 5 empyemas), 30 tuberculous pleural effusions (TPEs), 36 malignant pleural effusions (MPEs), and 20 transudative effusions. Pleural fluid NAMPT levels were highest in the patients with empyemas [575.4 (457.7, 649.3) ng/ml], followed by those with complicated PPEs [113.5 (103.5, 155.29) ng/ml], uncomplicated PPEs [24.9 (20.2, 46.7) ng/ml] and TPEs [88 (19.4, 182.6) ng/ml], and lower in patients with MPEs [11.5 (6.5, 18.4) ng/ml] and transudative effusions [4.3 (2.6, 5.1) ng/ml]. Pleural fluid NAMPT levels were significantly higher in PPEs (P < 0.001) or TPEs (P < 0.001) than in MPEs. Moreover, Pleural fluid NAMPT levels were positively correlated with the neutrophil percentage and lactate dehydrogenase (LDH) levels and inversely correlated with glucose levels in both PPEs and TPEs, indicating that NAMPT was implicated in the neutrophil-associated inflammatory response in infectious pleural effusion. Further, multivariate logistic regression analysis showed pleural fluid NAMPT was a significant predictor distinguishing PPEs from MPEs [odds ratio (OR) 1.180, 95% confidence interval (CI) 1.052–1.324, P = 0.005]. Receiver-operating characteristic (ROC) analysis demonstrated that NAMPT was a promising diagnostic factor for the diagnosis of infectious effusions, with the areas under the curve for pleural fluid NAMPT distinguishing PPEs from MPEs, TPEs from MPEs, and IPEs (PPEs and TPEs) from NIPEs were 0.92, 0.85, and 0.88, respectively. In conclusion, pleural fluid NAMPT could be used as a biomarker for the diagnosis of infectious pleural effusions.

## Introduction

A pleural effusion is a collection of excess fluid in the pleural cavity. Pleural infection is a common cause of pleural effusions in clinical practice, which encompass primary pleural infections without contiguous pneumonia, parapneumonic effusions (PPEs), and tuberculous pleural effusions (TPEs)^1^. As reported from a study with more than 3,000 patients with pleural effusions subjected to a diagnostic thoracentesis, PPEs and TPEs accounted for 19% and 9%, respectively^2^. And about 15–44% of patients with pneumonia may present with parapneumonic pleural effusion^3^. Clinical treatment for infectious (IPEs) and non-infectious pleural effusions (NIPEs) is entirely different, so misdiagnosis or delayed appropriate treatment can be detrimental to patients. However, in clinical practice, the accurate diagnosis of infectious pleural effusion is a challenge, especially if the pleural effusion is negative for Gram stain and bacterial/*Mycobacterium tuberculosis* cultures, often requiring extensive laboratory testing for possible etiology. Therefore, a potential accurate biomarker for the identification of infectious pleural effusion is of great clinical importance.

NAMPT, known as nicotinamide phosphoribosyltransferase (NAMPT) and Pre-B cell colony enhancing factor (PBEF), is considered as a proinflammatory cytokine that modulates the immune response^4^. Numerous studies support that NAMPT is essentially involved in inflammation. Its expression and secretion are increased in several inflammatory diseases such as acute lung injury, psoriasis^5^, acute chotecystitis^6^, and rheumatoid arthritis (RA)^7^. It is also believed to play a role in several types of infection like sepsis^8^, pneumonia^9^, and intrauterine infection (chorioamnionitis)^10^. However, it is unknown whether NAMPT plays a role in infectious pleural effusion.

In this study, we investigated the concentration of NAMPT in pleural effusions of various etiologies. Further studied the role of NAMPT in infectious pleural effusions and explored the clinical value of pleural fluid NAMPT in distinguishing infectious pleural effusions from non-infectious pleural effusions.

## Results

### General characteristics of the study population

A total of 111 patients with pleural effusion were included in this study, 25 parapneumonic effusions (17 uncomplicated PPEs, 3 complicated PPEs, and 5 empyemas), 30 tuberculous pleural effusions, 36 malignant pleural effusions (MPEs), and 20 transudative effusions (transudates). Of these patients, PPEs and TPEs were considered as infectious pleural effusions and MPEs as non-infectious pleural effusions. The demographic and clinical features and the pleural fluid characteristics of the patients are shown in Table 1.

**Table 1:** Clinical characteristics of the patients in the study.

Variable	Transudate (n = 20)	MPE (n = 36)	TPE (n = 30)	PPE total (n = 25)	PPE (n = 25)		
					UCPPE (n = 17)	CPPE (n = 3)	Empyema (n = 5)
Age (years)	83 (70.8, 89)	65 (61, 75.3)	42 (24, 60.8)^#^	67 (57, 83)	67 (57, 85)	78 (65,79)	65 (46.5, 79.5)
Gender (male/female)	13/7	23/13	23/7	20/5	14/3	3/0	3/2
Smoking	9 (45.0%)	12 (33.3%)	10 (33.3%)	10 (40.0%)	7 (41.1%)	2 (66.7%)	1 (20.0%)
Alcoholism	1 (5.0%)	8 (22.2%)	10 (33.3%)	9 (36.0%)	5 (29.4%)	2 (66.7%)	2 (40.0%)
**Major comorbidity**							
Congestive heart failure (n (%))	20 (100.0%)	2 (5.6%)	1 (3.3%)	4 (16.0%)	3 (17.6%)	0 (0.0%)	1 (20.0%)
Liver cirrhosis (n (%))	1 (5.0%)	1 (2.8%)	2 (6.7%)	0 (0.0%)	0 (0.0%)	0 (0.0%)	0 (0.0%)
Cerebrovascular disease (n (%))	2 (10.0%)	1 (2.8%)	0 (0.0%)	3 (12.0%)	2 (11.8%)	1 (33.3%)	0 (0.0%)
Hypertension (n (%))	10 (50.0%)	16 (44.4%)	6 (20.0%)#	11 (44.0%)	9 (52.9%)	0 (0.0%)	2 (40.0%)
Diabetes mellitus (n (%))	7 (35.0%)	3 (8.3%)	3 (10.0%)	2 (8.0%)	1 (5.9%)	0 (0.0%)	1 (20.0%)
COPD (n (%))	6 (30.0%)	2 (5.6%)	1 (3.3%)	1 (4.0%)	1 (5.9%)	0 (0.0%)	0 (0.0%)
**Pleural fluid**							
WBC (cells/μl)	570 (350, 1015)	1320 (712.5, 2275.5)	2305 (1355, 3500)#	3100 (1630, 7550)*	3000 (1630, 4765)	1460 (1400, 20,000)	7100 (4100, 92,400)
Neutrophils (%)	27 (13.5, 40)	24.5 (16.5, 45.0)	14.1 (10, 24.3)#	53 (31, 80)*	46 (30, 61.5)	58 (10, 85)	80 (70.5, 83.5)
Lymphocytes (%)	38.5 (25.5, 59.3)	54.5 (34.3, 62.3)	78.5 (66.5, 85)#	33 (12, 43)*	37 (17.5, 53)	10 (5, 85)	15 (11, 26)
Total protein (g/l)	23.9 (20.6, 25.3)	44.7 (38.2, 50.6)	51 (49.0, 54.4)#	32.5 (25.2, 47.3)	33.9 (28.6, 48.1)	46.1 (12.3, 47.7)	30.2 (23, 39.9)
Glucose (mmol/l)	7.4 (6.9, 9.5)	6.4 (5.7, 7.6)	5.3 (3.6, 6.3)#	6.05 (2.7, 4.9)	6.6 (5.8, 7.3)	0.34 (0.27, 2.61)	1.56 (0.4, 4.6)
ADA (U/l)	4.5 (3.0, 5.8)	8.5 (6.0, 11.3)	42 (33, 50)#	14 (9, 21)*	11 (7.5, 14)	24 (17, 43)	43 (28.5, 48.5)
LDH (U/l)	103 (87.3, 145.5)	259 (144, 380.5)	578.5 (366.8, 733)#	431 (211.5, 1283.5)*	254 (178, 464)	1753 (1202, 2073)	2362 (1080.5, 3395.5)
NAMPT (ng/ml)	4.3 (2.6, 5.1)	11.5 (6.5, 18.4)	88.2 (19.4, 182.6)#	45.8 (21.3, 134.4)*	24.9 (20.2, 46.7)	113.5 (103.5, 155.3)	575.4 (475.7, 649.3)

*MPE* malignant pleural effusion, *TPE* tuberculous pleural effusion, *PPE* parapneumonic effusion, *UPPE* uncomplicated parapneumonic effusion, *CPPE* complicated parapneumonic effusion, *COPD* chronic obstructive pulmonary disease, *WBC* White blood cell count, *ADA* adenosine deaminase, *LDH* lactate dehydrogenase, *NAMPT*, nicotinamide phosphoribosyltransferase; Data of CPPEs are presented as median (minimum, maximum); Other data are presented as median (interquartile ranges); ^#^p < 0.05 tuberculous vs. malignant pleural effusion; *p < 0.05 parapneumonic vs. malignant pleural effusion.

### Elevated pleural fluid NAMPT levels in IPEs

**Figure 1:**
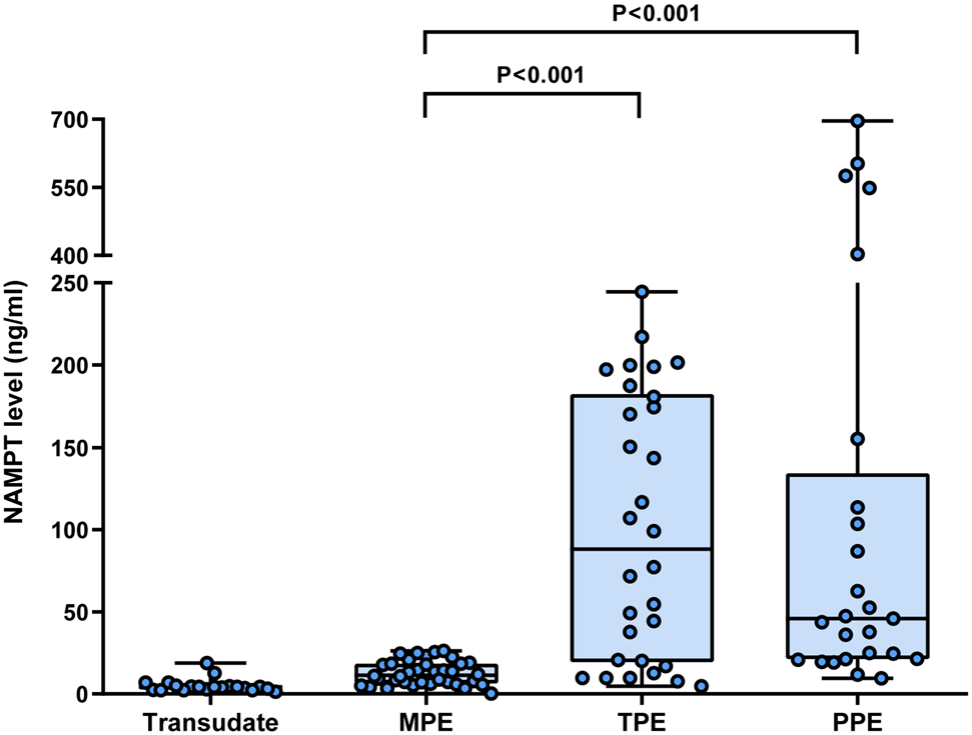
Distribution of pleural fluid NAMPT levels in the different diagnostic groups. *MPE* malignant pleural effusion, *TPE* tuberculous pleural effusion, *PPE* parapneumonic effusion.

The levels of pleural fluid NAMPT were analyzed by sandwich ELISA. Pleural fluid NAMPT levels in patients with PPEs, TPEs, MPEs and transudates were 45.8 (21.3, 134.4) ng/ml, 88.2 (19.4, 182.6) ng/ml, 11.5 (6.5, 18.4) ng/ml, and 4.3 (2.7, 5.1) ng/ml, respectively (Table 1). Pleural fluid NAMPT levels were found to be elevated in infectious pleural effusions (PPEs and TPEs). Patients with PPEs (P < 0.001) or TPEs (P < 0.001) had significantly higher pleural fluid NAMPT levels than those with MPEs (Fig. 1). Even empyema was excluded from PPEs, the NAMPT levels of PPEs were still higher than those of MPEs (P = 0.002).

**Figure 2:**
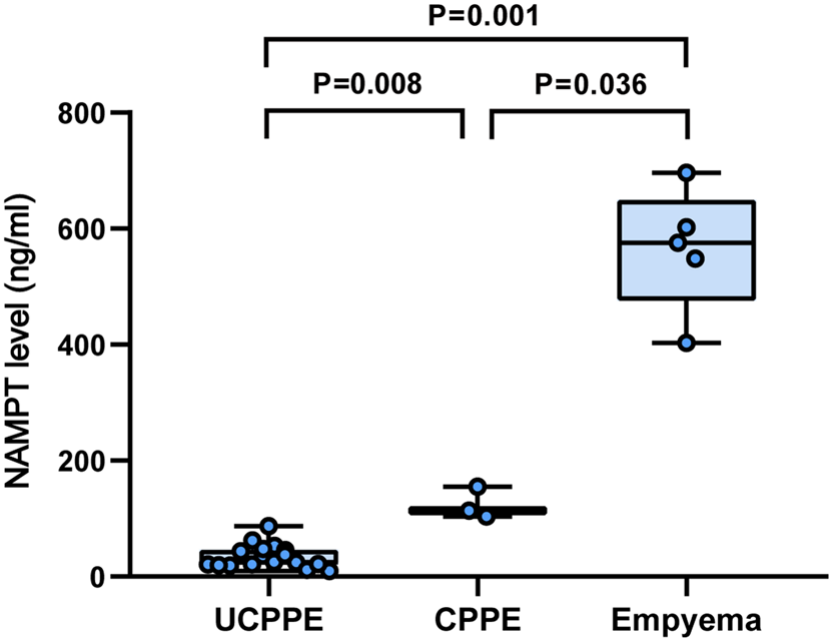
Pleural fluid NAMPT levels in patients with uncomplicated (UCPPE) and complicated (CPPE) parapneumonic pleural effusion and empyema.

To further investigate the release of NAMPT during the progression of parapneumonic effusion, we compared the pleural fluid NAMPT levels in patients with uncomplicated PPEs (UCPPEs), complicated PPEs (CPPEs), and empyemas (Fig. 2). Pleural fluid NAMPT levels in patients with UCPPEs, CPPEs, and empyemas were 24.9 (20.2, 46.7) ng/ml, 113.5 (103.5, 155.3) ng/ml, and 575.4 (457.7, 649.3) ng/ml, respectively. Pleural fluid NAMPT levels were significantly higher in patients with empyemas than in those with UCPPEs (P = 0.001) and CPPEs (P = 0.036), and higher in patients with CPPEs than those with UCPPEs (P = 0.008).

### Pleural fluid NAMPT levels are associated with markers of pleural inflammation in IPEs

**Figure 3:**
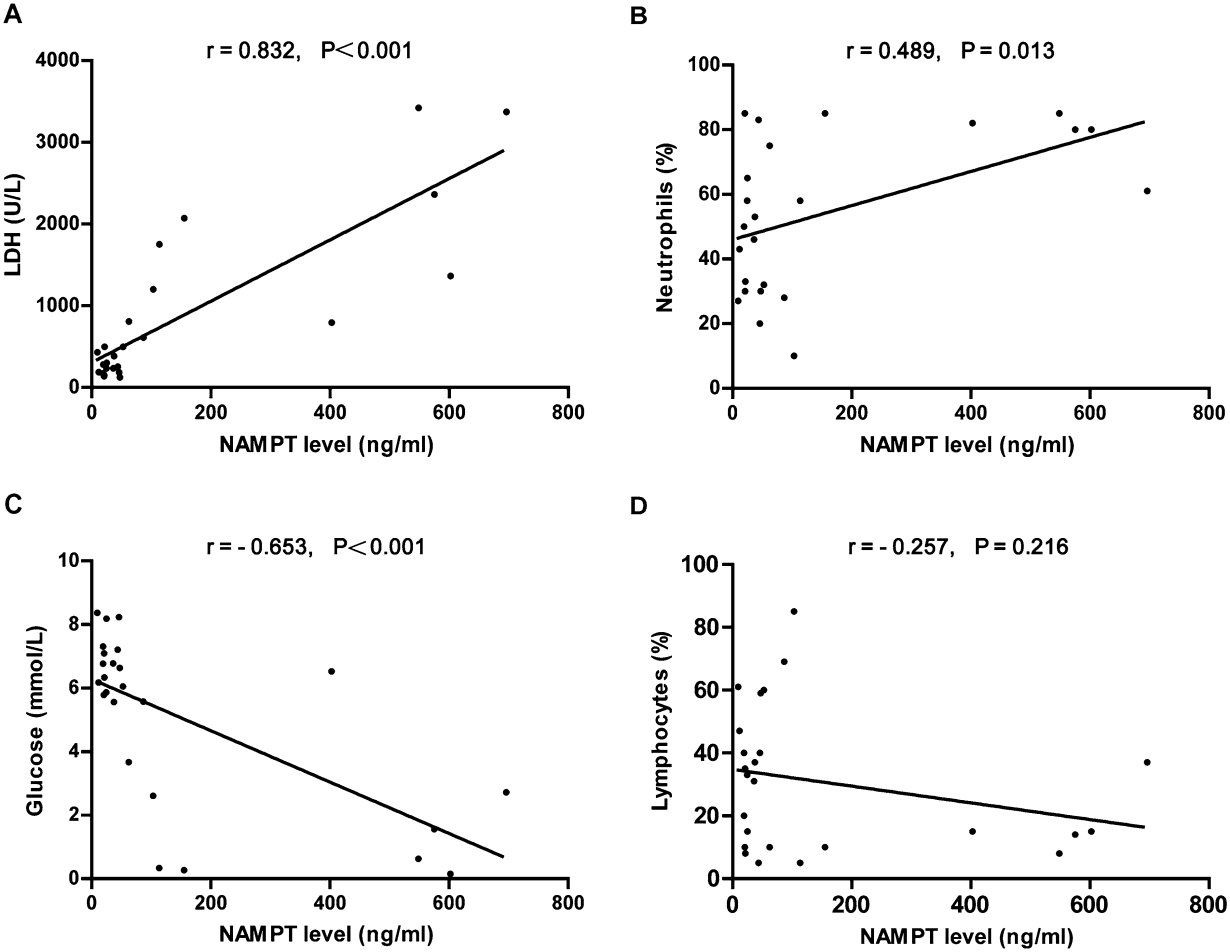
Correlation between pleural fluid NAMPT levels and LDH levels, neutrophil percentage, glucose levels, and lymphocyte percentage in parapneumonic effusions.

**Figure 4:**
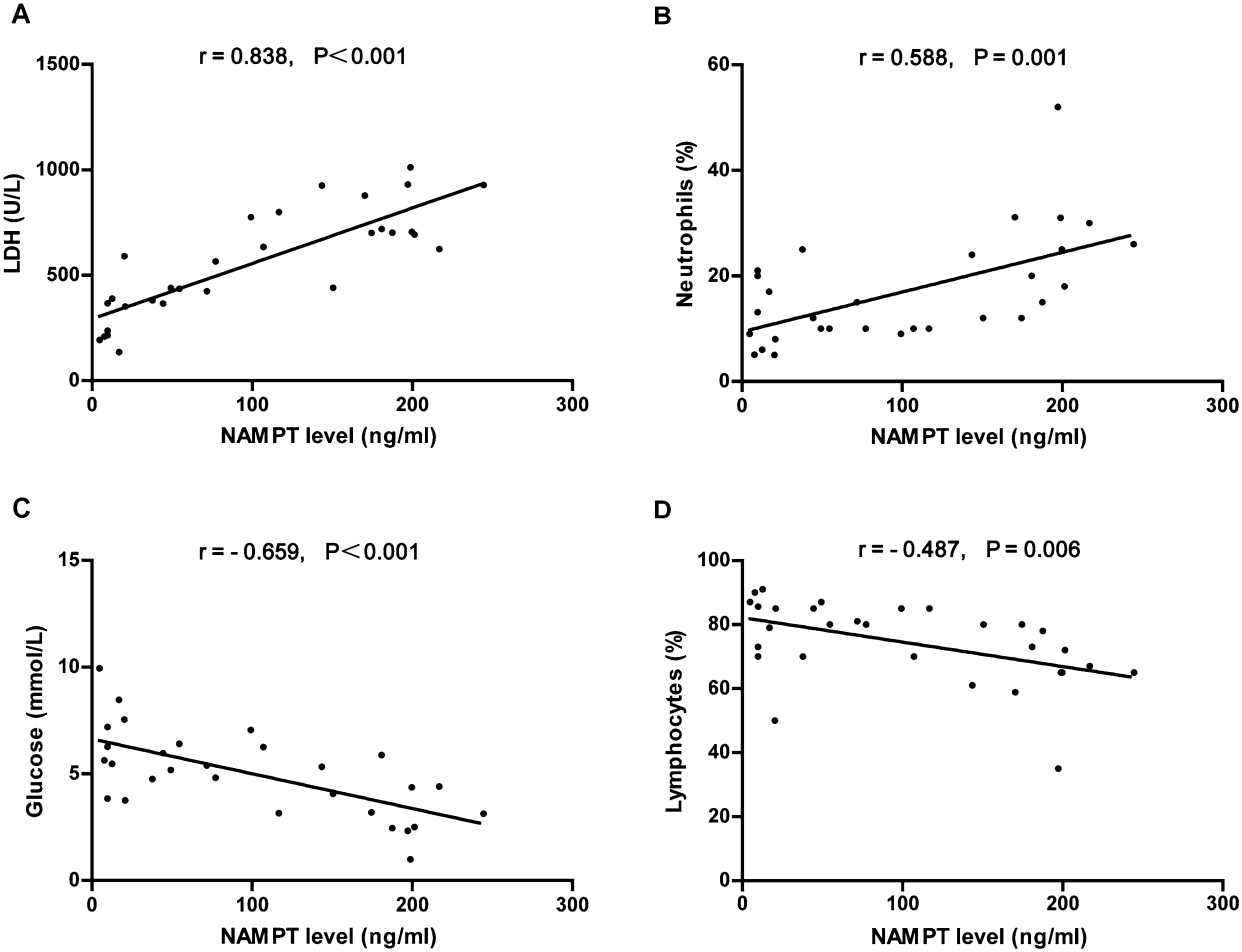
Correlation between pleural fluid NAMPT levels and LDH levels, neutrophil percentage, glucose levels, and lymphocyte percentage in tuberculous pleural effusions.

Considering the increased NAMPT levels in the PPE and TPE groups, we next assessed the relationship between pleural fluid NAMPT levels and the markers of pleural inflammation. Pleural fluid NAMPT levels were positively correlated with the acute phase reactant lactate dehydrogenase (LDH) levels (r = 0.832, P < 0.001) and pleural fluid neutrophil percentage (r = 0.489, P = 0.013), and inversely correlated with glucose levels (r = − 0.653, P < 0.001) and pleural fluid lymphocyte percentage (r = − 0.257, P = 0.216) in PPE group (Fig. 3). Similarly, pleural fluid NAMPT levels of TPE were positively correlated with pleural LDH levels (r = 0.838, P < 0.001) and pleural fluid neutrophil percentage (r = 0.588, P = 0.001), and inversely correlated with glucose levels (r = − 0.659, P < 0.001) and the percentage of pleural fluid lymphocyte percentage (r = − 0.487, P = 0.006; Fig. 4).

### Univariate analyses and multivariate logistic regression analyses for diagnosis of IPEs

Univariate analyses were performed to estimate each factor between PPEs and MPEs, and TPEs and MPEs (Table 1). Variables that were statistically significant in the univariate analysis and considered clinically relevant were further analyzed by multivariate logistic regression analysis (Table 2). Given the number of events available, variables were carefully selected for inclusion to ensure parsimony of the final model. In the multivariate logistic analysis, pleural fluid NAMPT levels remained associated with increased diagnostic odds for the identification of PPEs from MPEs [odds ratio (OR) 1.180, 95% confidence interval (CI) 1.052–1.324, P = 0.005]. However, no statistical significance of pleural fluid NAMPT on identifying TPEs versus MPEs was found by multivariate analysis, although the univariate analysis was statistically significant.

**Table 2:** Multivariate logistic regression analysis for distinguishing PPEs or TPEs from MPEs.

Parameter	P value	OR	95% CI
**Distinguishing PPEs from MPEs**			
Pleural fluid WBC(cells/μl)	0.154	1.000	1.000–1.001
Pleural fluid neutrophils (%)	0.531	1.014	0.970–1.060
Pleural fluid LDH (U/l)	0.326	0.996	0.989–1.004
Pleural fluid NAMPT (ng/ml)	0.005	1.180	1.052–1.324
**Distinguishing TPEs from MPEs**			
Pleural fluid ADA (U/l)	0.033	1.432	1.030–1.991
Pleural fluid lymphocytes (%)	0.306	1.099	0.917–1.318
Pleural fluid NAMPT (ng/ml)	0.648	1.034	0.896–1.194

*PPE* parapneumonic effusion, *TPE* tuberculous pleural effusion, *MPE* malignant pleural effusion, *WBC* white blood cell count, *LDH* lactate dehydrogenase, *NAMPT* nicotinamide phosphoribosyltransferase, *ADA* adenosine deaminase, *OR* odds ratio, *CI* confidence interval.

### Diagnostic value of pleural fluid NAMPT for IPEs

**Figure 5:**
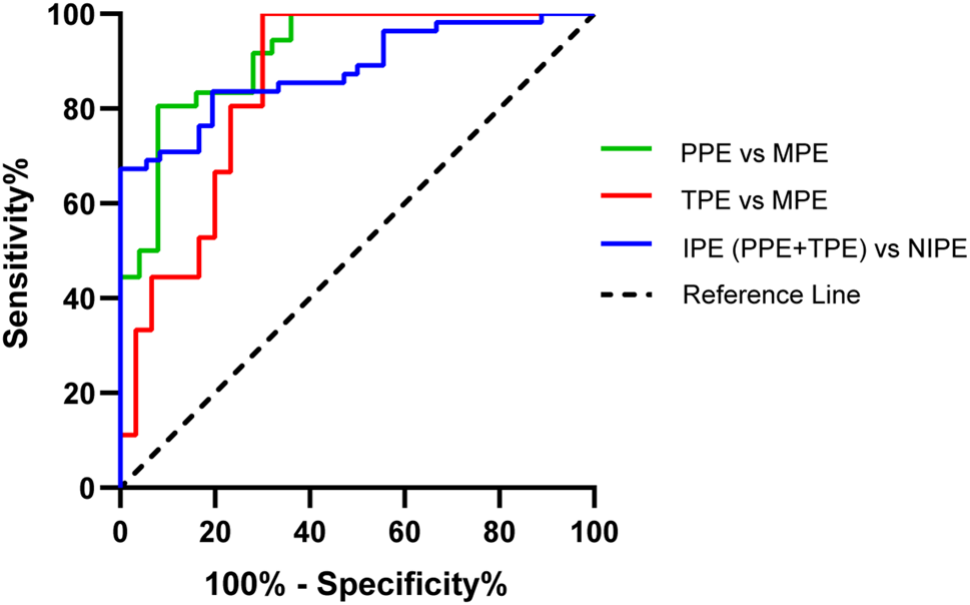
Receiver operating characteristics (ROC) curves of pleural fluid NAMPT for distinguishing infectious pleural effusions. *PPE* parapneumonic effusion, *TPE* tuberculous pleural effusion, *MPE* malignant pleural effusion, *IPE* infectious pleural effusion, *NIPE* non-infectious pleural effusion.

The efficacy of pleural fluid NAMPT in distinguishing PPEs from MPEs, TPEs from MPEs, and IPEs (PPEs and TPEs) from NIPEs were evaluated by assessing receiver-operating characteristic (ROC) analysis with areas under the curve (AUCs) of 0.92, 0.85, and 0.88, respectively (Fig. 5 and Table 3). At a cut-off value of 19.11 ng/ml, pleural fluid NAMPT had a sensitivity of 80.56%, specificity of 92%, positive predictive value (PPV) of 79.31%, negative predictive value (NPV) of 93.75%, and accuracy of 87% for discriminating PPEs from MPEs. At the cut-off of 31.93 ng/ml, pleural fluid NAMPT had a sensitivity of 70%, specificity of 100%, PPV of 100%, NPV of 80%, and accuracy of 86% for discriminating TPEs from MPEs. And at the cut-off of 19.11 ng/ml, pleural fluid NAMPT had a sensitivity of 86.11%, specificity of 80%, PPV of 87.04%, NPV of 78.38%, and accuracy of 84% for discriminating IPEs (PPEs and TPEs) from NIPEs.

**Table 3:** Diagnostic performance of pleural fluid NAMPT based on the receiver operator characteristic (ROC) analysis.

	Cut-off point (ng/ml)	Sensitivity (%)	Specificity (%)	PPV (%)	NPV (%)	Accuracy (%)	AUC
PPE vs. MPE	> 19.11	80.56	92.00	79.31	93.75	87.00	0.92
TPE vs. MPE	> 31.93	70.00	100.00	100.00	80.00	86.00	0.85
IPE (PPE + TPE) vs. NIPE	> 19.11	86.11	80.00	87.04	78.38	84.00	0.88

*PPE* parapneumonic effusion, *MPE* malignant pleural effusion, *TPE* tuberculous pleural effusion, *IPE* infectious pleural effusion, *NIPE* non-infectious pleural effusion, *PPV* positive predictive value, *NPV* negative predictive value, *AUC* area under the curve.

## Discussion

The main finding of the study was the increased NAMPT levels in infectious pleural effusions (PPEs and TPEs). On etiology-based comparison of pleural effusion, pleural fluid NAMPT levels were observed highest in the patients with empyemas, followed by those with TPEs or UPPEs or CPPEs, and NAMPT concentrations were lower in patients with MPEs and transudative effusions. Moreover, NAMPT was implicated in the neutrophil-associated inflammatory response in PPEs and TPEs. Further, pleural fluid NAMPT was found to be an independent predictor for differentiating PPEs from MPEs and had good diagnostic value for infectious pleural effusions.

Our study found that NAMPT levels were elevated in infectious pleural effusions, which was consistent with the reports of NAMPT in infectious disease. Elevated plasma NAMPT had been reported in patients with infectious diseases, such as sepsis, pneumonia, COPD, and acute lung injury^8,9,11,12^. Meanwhile, NAMPT levels were also found to correlate with inflammatory cytokines or mediators in patients with these diseases and associate with the degree of inflammation and disease severity. A previous study enrolled 102 patients with severe sepsis and 102 healthy controls, and serum NAMPT was significantly higher in patients with sepsis than controls and higher in patients with septic shock^8^. In patients with community-acquired pneumonia (CAP), peripheral plasma NAMPT levels were elevated and associated with inflammation markers, including blood leukocytes and C-reactive protein in the acute phase; NAMPT was believed involved in the inflammatory process and associated with CAP severity^9^.

According to the pathogenesis, we classified parapneumonic pleural effusions into three types or three stages: uncomplicated PPE, complicated PPE, and empyema. Parapneumonic effusions are initially sterile and free-flowing effusions. As bacteria invade, pleural inflammation progressively increases. Multiple proinflammatory factors stimulate neutrophils for migration and fibrocytes for chemotaxis, accompanied by an increase in pleural lactic dehydrogenase (LDH) and a decrease in pleural glucose and pH, which can eventually develop into a CPPE or even empyema^13^. Our finding was that pleural NAMPT levels were markedly higher in empyemas than in CPPEs and UCPPEs, and higher in CPPEs than in UCPPEs, along with a positive correlation between pleural fluid NAMPT levels and neutrophil percentage and LDH levels, and an inverse correlation with glucose levels, indicating that NAMPT might be implicated in the escalation of parapneumonic effusions. Also, NAMPT was thought to be involved in the neutrophil-associated inflammatory response in PPEs. Compared to patients with UCPPE, those with empyema or CPPE may have a more prolonged course and a potentially higher mortality rate. In this regard, NAMPT may have great potential in differentiating the stages of PPEs and assisting clinical decision-making, as NAMPT was correlated with the disease severity of PPEs.

Tuberculous pleural effusions are thought to trigger a local immune response by rupture of a subpleural focus of pulmonary disease and release of small amounts of *Mycobacterium tuberculosis* into the pleural space, starting with an influx of neutrophils, followed by monocyte migration and a strong lymphocyte response^14^. In TPEs, we also observed an increase in NAMPT levels. NAMPT levels in TPEs were positively correlated with neutrophil percentage and LDH levels and negatively correlated with lymphocyte percentage and glucose levels. We hypothesize that the early stage of TPE is dominated by neutrophils accompanied by a rapidly elevated NAMPT level; as TPE progresses, lymphocytes accumulate and proliferate in the inflamed pleural space, subsequent decrease in the NAMPT level. Like in PPEs, NAMPT may also be primarily involved in the neutrophil-associated inflammatory response in tuberculous pleural effusions, especially in the early stage of TPEs.

However, the biological function and pathogenic mechanisms of high levels of NAMPT in infectious pleural effusion are still unknown. Infectious pleural effusion is a complex environment containing a variety of proteins and immune cells. Activated immune cells had been proved to release NAMPT, including neutrophils, monocytes, and macrophages^10^. NAMPT was found to be induced by several inflammatory mediators following inflammatory stimuli, including lipopolysaccharide (LPS), IL-1β, IL-6, and TNF-α^4,15^. It was also shown to increase inflammatory cytokines, such as TNF-α, IL-1β, IL-16, TGF-β1, and the chemokine receptor CCR3. Moreover, NAMPT was previously proved instrumental in inhibiting apoptosis of neutrophils during the inflammation period^16^, which was in line with our finding of a positive correlation between NAMPT levels and neutrophil percentage in PPEs and TPEs. Thus, we speculate that the release of NAMPT is triggered by inflammation markers in infectious pleural effusions and prevents neutrophil apoptosis to sustain/increase the inflammatory response. However, it is worthwhile to elucidate the mechanism of augmented NAMPT in infectious pleural effusion in the future.

Pleural fluid NAMPT was the only significant predictor distinguishing PPEs from MPEs in the multivariate regression analysis (OR 1.180, 95% CI 1.052–1.324, P = 0.005). Other parameters such as white blood cell count (WBC), neutrophil percentage, and lactate dehydrogenase reached statistical significance in the univariate analysis but not in the multivariate analysis. Using ROC analysis, pleural fluid NAMPT of 19.11 ng/ml had a sensitivity of 80.56%, specificity of 92%, PPV of 79.31%, NPV of 93.75%, accuracy of 87%, and AUC of 0.92 for discriminating PPEs from MPEs. In identifying TPEs from MPEs, pleural fluid NAMPT does not appear to have an advantage over adenosine deaminase (ADA), given that the result of the multivariate analysis showed that only ADA had an independent predictive effect for TPEs. Although multivariate logistic regression analysis negated the independent role of NAMPT, it still had the diagnostic ability to distinguish TPEs from MPEs. At the cut-off of 31.93 ng/ml, pleural fluid NAMPT had a sensitivity of 70%, specificity of 100%, PPV of 100%, NPV of 80%, accuracy of 86%, and AUC of 0.85 for discriminating TPEs from MPEs. In addition, we also demonstrated the high diagnostic power of NAMPT for infectious pleural effusions (PPEs and TPEs) by ROC analysis. At the cut-off of 19.11 ng/ml, pleural fluid NAMPT had a sensitivity of 86.11%, specificity of 80%, PPV of 87.04%, NPV of 78.38%, and accuracy of 84% for discriminating IPEs (PPEs and TPEs) from NIPEs.

Compared with the acute reactant LDH, NAMPT appears to be superior to LDH in identifying infectious pleural effusions. The current study demonstrated that NAMPT was exclusively elevated in infectious pleural effusions. LDH is an acute reactant associated with the degree of inflammation in infectious pleural effusions; however, it is also elevated in malignant pleural effusions as LDH also acts as an intracellular enzyme that indicates the degree of cell turnover within the pleural space^1^. In addition, several studies have explored the potential utility of other new biomarkers of infection (e.g., C-reactive protein^17^, procalcitonin^18,19^, presepsin^20^, cell-free DNA^21^, Interleukin-27^22^) to diagnose infectious pleural effusion. A prior study reported 75 patients with significantly elevated pleural fluid procalcitonin in IPEs (empyemas and PPEs) and pleural fluid procalcitonin > 0.25 ng/ml had a sensitivity of 77.78% and specificity of 74.14% for the diagnosis of IPEs^19^. Another retrospective study including 132 patients found that pleural fluid CRP > 2.59 mg/dl had a 65.8% sensitivity and 90.4% specificity, procalcitonin > 0.11 ng/ml had a 63.2% sensitivity and 74.5% specificity, and presepsin > 680 pg/ml had a 68.4% sensitivity and 74.5% specificity for the diagnosis of IPEs (PPEs and TPEs)^20^. Compared to these new biomarkers, pleural fluid NAMPT could be used to differentiate PPEs from MPEs, TPEs from MPEs, and IPEs from NIPEs with better sensitivity and specificity.

In addition, some complementary contents need to be illustrated. First, LDH was lower in the PPE group than in the TPE group, which seems inconsistent with studies by others. We explain that patients in the TB group were younger than those in the pneumonia group, that some patients with PPE had received antimicrobial drugs before admission or chest drainage. Second, the sample sizes for CPPE and empyema were too small to allow us to compare them as separate groups with the MPE group. Third, pleural fluid pH is supposed to be an important parameter for the diagnosis of CPPE, but we did not obtain the data as our laboratory department does not routinely conduct pH tests for pleural effusions. Last but not least, blood samples were not collected from patients in our study, so we were unable to compare NAMPT levels in serum and pleural effusions in patients with pleural effusions.

In conclusion, we report that NAMPT levels were elevated in infectious pleural effusions (PPEs and TPEs). And we elucidated the involvement of NAMPT in the neutrophil-associated inflammatory response in infectious pleural effusions and clarified that NAMPT might be a novel and promising biomarker for identifying infectious pleural effusions. However, continued studies are needed to further explore the role of NAMPT in pleural effusions.

## Materials and methods

### Participants

This prospective study was conducted in the Department of Pulmonary and Critical Care Medicine of the Affiliated Hospital of Medical School of Ningbo University from January 1, 2016, to November 6, 2020. A chest X-ray, chest computerized tomography (CT) scan, or chest sonography was used to screen patients for pleural effusions. Patients with pleural effusions and planned thoracentesis were initially enrolled in the study. Those patients with an unclear diagnosis of pleural effusion were excluded from further participation. Finally, a total of 111 patients with pleural effusion were included in the study.

Of these patients with a definite diagnosis of pleural effusion, there were diagnoses of parapneumonic effusions, tuberculous pleural effusions, malignant pleural effusions, and transudative effusions. Parapneumonic effusions were effusions associated with bacteria pneumonia, lung abscesses, bronchiectasis, or the presence of pus in the pleural space. Parapneumonic effusions were further classified as uncomplicated PPEs, complicated PPEs, and empyemas. CPPEs referred to effusions with glucose < 3.3 mmol/l (< 60 mg/dl) and LDH > 1000 U/l. Empyemas referred to collections of pus within the pleural space. Tuberculous pleural effusions were diagnosed with evidence of the following criteria: positive culture for *Mycobacterium tuberculosis* in sputum, pleural effusion, pleural biopsy, or bronchial aspirate; the presence of typical epithelioid cell granuloma on pleural biopsy by histologic examination; lymphocytic predominant exudate with high pleural fluid ADA level; clinical and laboratory data suggestive of tuberculosis or effective with anti-TB treatment. The diagnosis of malignant pleural effusions was based on cytological analyses confirming malignant cells or imaging-guided biopsies demonstrating MPEs, or the patient already had a confirmed malignant disease and no other cause of the effusion. Transudates were effusions that met the Light’s criteria and in our study were all secondary to heart failure^23^. In this study, parapneumonic effusions and tuberculous pleural effusions were classified as infectious pleural effusions, and malignant pleural effusions were classified as non-infectious pleural effusions.

The study was approved by the Ethics Committee of the Affiliated Hospital of Medical School of Ningbo University. All patients signed informed consent of undergoing thoracocentesis and the acquisition of human samples. The research was performed in accordance with the relevant guidelines and regulations.

### Pleural fluid collection and routine examination

Pleural fluid specimens were collected from all the patients conducted by thoracentesis. Pleural fluid samples were immediately subjected to routine examinations for analysis, including total and differential cell counts, protein, glucose, lactate dehydrogenase, ADA, and cytological and microbiological tests in pleural effusion. The remaining pleural effusions were collected and centrifuged at 2000×*g* for 5 min immediately at 4 °C. Cell-free supernatants were stored at − 80 °C for late detection.

### Pleural effusion NAMPT levels measurement

The samples of the pleural effusion were defrosted at 4 °C in a room. NAMPT concentrations in the pleural fluid were assessed by enzyme-linked immunosorbent assay (ELISA Kit: AdipoGen; San Diego, USA), and the detection was conducted according to the manufacturer’s instructions. All measurements were carried out with the operator blinded to clinical data.

### Data analysis

The statistical analysis was performed using GraphPad Prism software (version 7; GraphPad Software, Inc.) and IBM SPSS Statistics software (version 24; IBM Corp.). Comparisons were performed in the univariate analyses using the following tests: t-tests for normally distributed variables, chi-square tests for categorical variables, and Mann–Whitney U tests for continuous non-normally distributed variables. Variables that were statistically significant in the univariate analysis and considered clinically relevant were entered into a multivariate logistic regression model. The goodness of fit of the model was assessed by Hosmer and Lemeshow tests. The ability of NAMPT to distinguish PPEs from MPEs, TPEs from MPEs, IPEs from NIPEs were evaluated using receiver-operating characteristic (ROC) analysis. Correlation analysis was used with the Spearman rank-order correlation to measure the associations between NAMPT and inflammation markers in pleural effusion. A P < 0.05 was considered to be significant. Data of CPPEs were presented as median (minimum, maximum); Other data were presented as median (interquartile ranges).

### Ethical approval

The study was approved by the Ethics Committee of the Affiliated Hospital of Medical School of Ningbo University.

## Data Availability

The datasets generated during and/or analysed during the current study are available from the corresponding author on reasonable request.
